# Difficulties in Diagnosing Gastric Adenocarcinoma of the Cervix: A Clinical Case and Literature Review

**DOI:** 10.7759/cureus.104304

**Published:** 2026-02-26

**Authors:** Eduardo Gonzalez-Bosquet, Santiago Gonzalez, Laia Ortiz Peidro, Sousa Cacheiro Sousa Cacheiro, Leornardo A Rojo Muchacho

**Affiliations:** 1 Gynecology, Sant Joan de Deu, Barcelona, ESP; 2 Gynecology, Sant Joan de Déu, Barcelona, ESP; 3 Diagnostic Radiology, Sant Joan de Deu, Barcelona, ESP; 4 Pathology, Sant Joan de Deu, Barcelona, ESP

**Keywords:** cervical cancer diagnosis, gastric-type mucinous carcinoma, human papilloma virus dna, treatment of cervical cancer, uterine cervical cancer

## Abstract

We present the case of a 58-year-old patient diagnosed with gastric-type endocervical adenocarcinoma, a rare type of cervical cancer not associated with human papillomavirus (HPV) infection. The diagnosis was prompted by the incidental finding of endometrial thickening (9 mm) during a routine check-up for urinary incontinence. Hysteroscopic endometrial biopsy raised suspicion for malignancy, and further evaluation with serum CA 19-9 tumor marker and computed tomography confirmed the cervical origin of the tumor. The disease was diagnosed at an advanced stage, and despite primary treatment with chemoradiotherapy, the tumor recurred. We also provide a review of the literature focusing on the diagnostic challenges, treatment strategies, and prognosis of this rare form of cervical cancer.

## Introduction

Gastric-type endocervical adenocarcinoma is a rare form of cervical cancer not associated with human papillomavirus (HPV) infection [1]. Endocervical carcinomas represent 20-25% of cervical cancers, with increasing incidence in recent years [2]. The most common type of cervical adenocarcinoma is HPV-associated, most frequently related to HPV genotypes 18 and 16 [3].

The World Health Organization (WHO) classification introduced a two-tiered system for endocervical adenocarcinomas based on HPV status, distinguishing between HPV-associated and HPV-independent tumors [4]. The concept of gastric-type cervical adenocarcinoma (GCA) was first described in 2007, when HIK1083 immunohistochemistry expanded the morphological spectrum of endocervical adenocarcinoma by identifying gastric differentiation beyond minimal deviation adenocarcinoma, which represents its well-differentiated subtype [5]. GCA was included as a variant of mucinous carcinoma in the 2014 WHO classification [6], and it is currently recognized as the most frequent HPV-independent endocervical adenocarcinoma variant [7-9].

In this report, we present a case of gastric-type endocervical adenocarcinoma and discuss the diagnostic challenges, treatment, and prognosis of this rare entity, together with a review of the literature.

## Case presentation

A 58-year-old patient consulted for urinary incontinence, with no other symptoms of interest. During the vaginal ultrasound examination, an endometrial thickness of approximately 6 mm was detected, which was considered abnormal in a postmenopausal patient. An attempt was made to perform an endometrial biopsy using a Cornier cannula; however, insufficient material was obtained. Endometrial cytology was subsequently performed, yielding a non-suspicious but diagnostically insufficient result.

The patient’s medical history included migraine, laparoscopic cholecystectomy, adenoidectomy, and two cesarean deliveries. She had no history of sexually transmitted diseases, used condoms as a method of contraception, and had regular Pap smears, the results of which were normal. She has no toxic habits and entered menopause at the age of 54. Her family history was notable for breast cancer in her sister and grandmother, both diagnosed after the age of 50.

**Figure 1:**
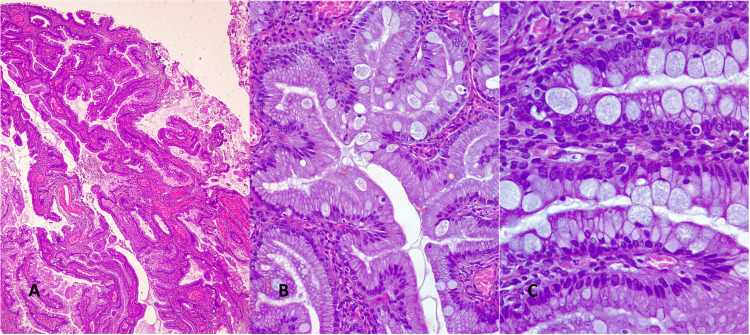
Histology biopsy of the first hysteroscopy A. Shows a well-differentiated pyloric-type mucinous adenocarcinoma (Gastric type endocervical adenocarcinoma) with an infiltrative growth pattern (H&E, 10×). B. Shows a well-differentiated, multibranching atypical glands exhibiting pyloric-type epithelial differentiation (Gastric type endocervical adenocarcinoma) with scattered goblet cells and minimal cytologic atypia (H&E, 20×). C. A 40× view highlighting atypical glands with pyloric-type epithelial differentiation (Gastric type endocervical adenocarcinoma) and dispersed goblet cells (H&E, 40×).

Given the absence of previous vaginal bleeding or any type of vaginal discharge, the endometrium was monitored for five months. Follow-up transvaginal ultrasound showed an increase in endometrial thickness to 9.3 mm, prompting diagnostic hysteroscopy with endometrial biopsy. Hysteroscopy revealed a polypoid lesion measuring approximately 2 cm with an implantation base on the anterior and fundal uterine surface, which was removed using a resectoscope. Histological examination showed a polypoid lesion with thick vascular structures and mucinous epithelium, raising a differential diagnosis between an endocervical polyp and a low-grade proliferative lesion. Immunohistochemical analysis showed negative p53 expression and no abnormalities in the DNA mismatch repair proteins (Figure 1). Due to diagnostic uncertainty, repeat evaluation of the uterine cavity was recommended.

 A second hysteroscopy was performed one month later, revealing a polypoid lesion with an implantation base in the cervical canal measuring approximately 9 mm. The lesion was removed and sent for histological analysis, along with a curettage of the endometrial cavity and endocervical canal.

**Figure 2:**
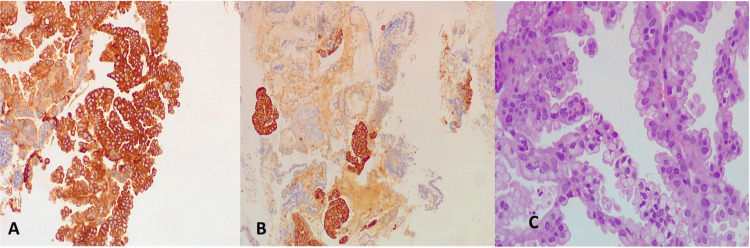
Mucinous tumor: CK7 and CK20 positive A. Shows strong membranous and cytoplasmic positivity for cytokeratin 7 in the gland-forming gastric-type endocervical adenocarcinoma (immunohistochemistry for CK7, 10×). B. Shows strong membranous and cytoplasmic positivity for CK20 in the gastric-type endocervical adenocarcinoma (immunohistochemistry for CK20, 10×). C. Image shows mucinous epithelium with minimal cytologic atypia (H&E, 20×).

Histology showed an endocervical polyp, while both the endocervical and endometrial curettage specimens revealed a mucinous neoplasm with a high cell proliferation index according to the Ki67 antigen. The tumor exhibited a complex architectural pattern, and the mucinous epithelium was positive for cytokeratin 7 (CK 7) and cytokeratin 20 (CK 20) (Figure 2), suggesting an ovarian genitourinary origin, without excluding other primary sites.

**Figure 3:**
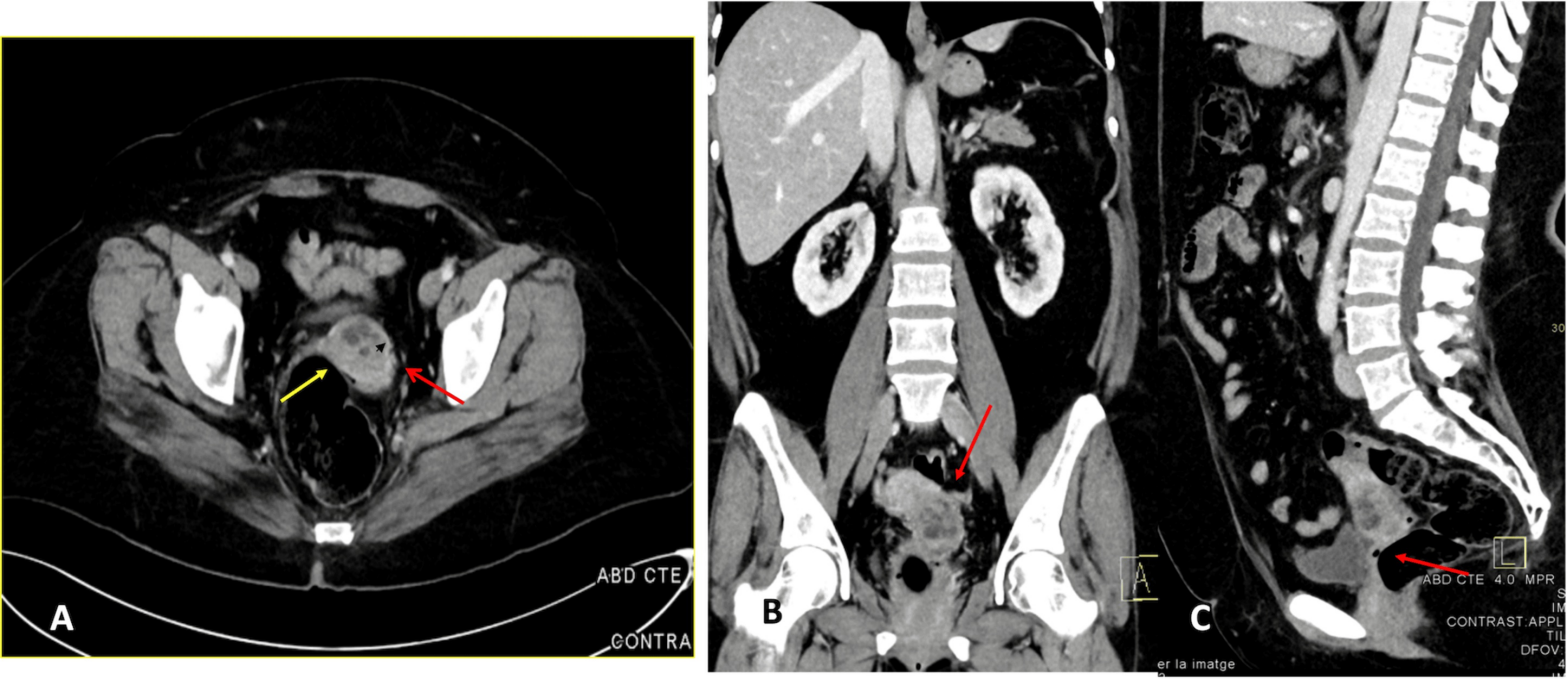
Computed tomography (CT) A. Axial CT+C portal venous phase CT features a soft tissue mass with heterogeneous enhancement (measuring 44x40 mm) centered on the uterine cervix, located between the yellow and red arrow. No abnormal pelvic or para-aortic lymph nodes. B. Coronal CT + portal venous phase. It appears to invade the uterine cavity, indicated by the red arrow. C. Coronal CT + portal venous phase. There is no apparent extension to the anterior rectal wall or the posterior wall of the bladder, as indicated by the red arrow.

As the primary origin of the neoplasm remained uncertain, an extension study was performed. Tumor markers and computed tomography (CT) were requested due to concern for an ovarian primary tumor. The CT imaging revealed a 44x40 mm tumor lesion affecting the uterine cervix with signs of infiltration to the uterine body and vaginal vault (Figure 3). Tumor markers, including carcinoembryonic antigen (CEA), CA-125, and HE-4, were within normal limits, while CA 19-9 was elevated at 178 U/ml (normal <37 U/ml).

**Figure 4:**
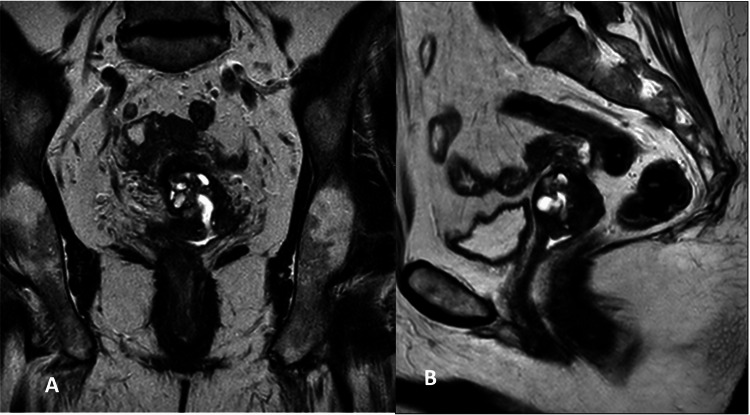
Magnetic resonance (MR) A. Coronal T2. Shows a well-defined T2 hyperintense multicystic lesion involving the cervix, containing variably sized solid portions. B. Sagittal T2. Shows no apparent extension to the anterior rectal wall or posterior bladder wall.

Magnetic resonance imaging (MRI) demonstrated a poorly delineated solid-cystic lesion consistent with adenocarcinoma of the cervix, with suspected parametrial infiltration predominantly on the left side and involvement of the upper third of the vagina (Figure 4).

**Figure 5:**
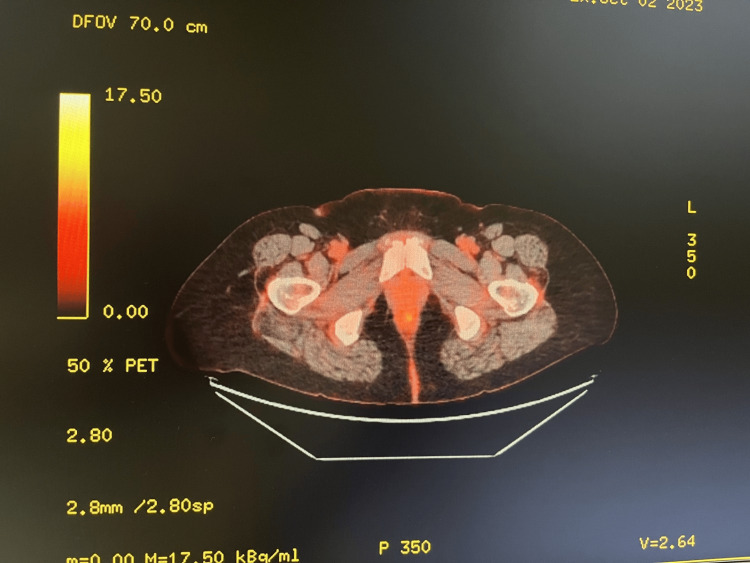
Positron emission tomography-computed tomography (PET-CT) Transverse plane. Cervical tumor with heterogeneous, mildly increased metabolic activity

An extension study was performed with positron emission tomography-computed tomography (PET-CT), which confirmed a cervical tumor with heterogeneous, mildly increased metabolic activity extending to the posterior uterine margin and vaginal vault, in contact with the rectum, without evidence of distant disease (Figure 5). No other findings of interest were observed.

**Figure 6:**
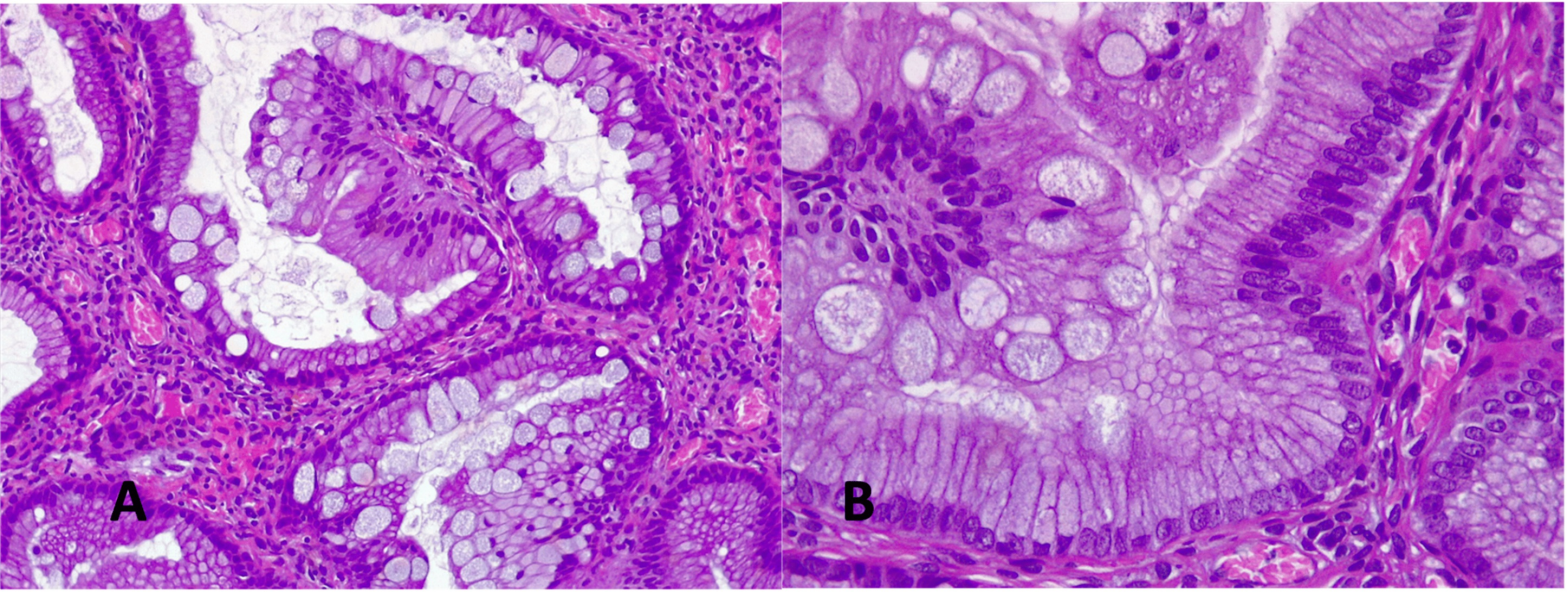
Cervical biopsy A. Mucinous-type epithelium (H&E, 20x) B. Mucinous-type epithelium (H&E, 40x)

**Figure 7:**
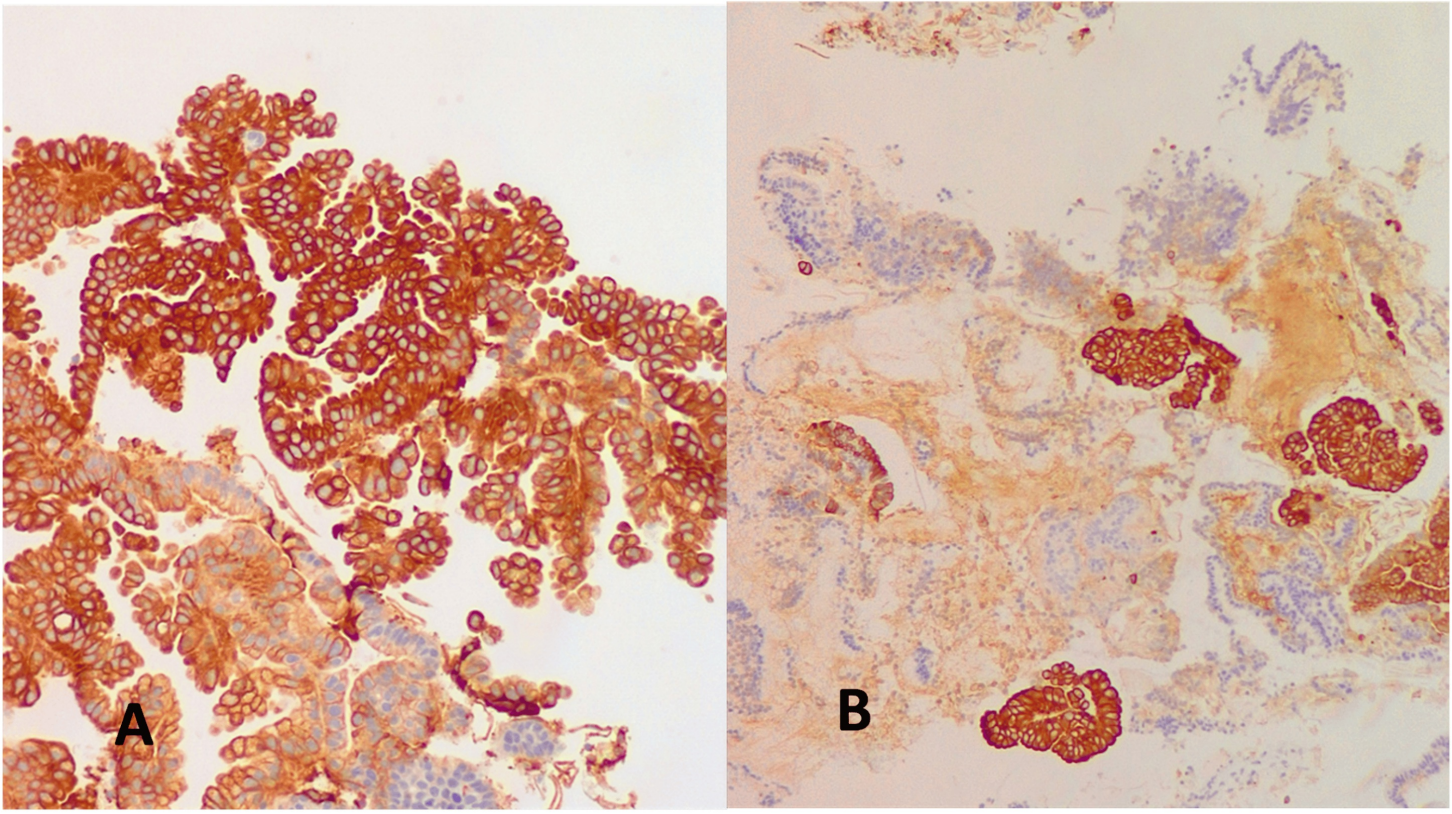
Immunohistochemically A. Positive for cytokeratin 7 (CK 7, 40x) B. Focal positivity for cytokeratin 20 (CK 20, 40x)

Histological review of the cervical biopsy showed glandular proliferation with a complex architectural pattern, lined by mucinous-type epithelium composed of tall cells with well-defined cytoplasmic borders, clear cytoplasm, and basally located nuclei with mild cytologic atypia. Goblet cells were present, with occasional mitotic figures (Figure 6). Immunohistochemically, the lesion was positive for CK7 and carbonic anhydrase IX (CAIX), with focal positivity for CK20 and PAX8 (Figure 7). MUC-6 was positive, and CEA showed apical staining. CDX2, estrogen receptors, and p16 were negative, while p53 exhibited a wild-type expression pattern.

Based on the morphological and immunohistochemical findings, the diagnosis of HPV-independent gastric-type cervical adenocarcinoma (WHO 2020 classification) was established [10].

The case was discussed at the multidisciplinary oncology committee,and a lymph node extension study was recommended. Para-aortic lymphadenectomy was performed, with no evidence of lymphatic extension. Primary treatment with chemoradiotherapy (CRT) was administered for stage IIB cervical cancer.

One year and three months after completion of treatment, a follow-up MRI scan revealed uterine recurrence, which was confirmed histologically by biopsy. PET-CT showed no evidence of distant metastasis, and robotic salvage hysterectomy was planned. During surgery, peritoneal metastases were identified and confirmed on biopsy, leading to abandonment of the surgical procedure. The multidisciplinary team recommended radiotherapy combined with immunotherapy (platinum-based chemotherapy plus pembrolizumab). Currently, four months after treatment of the recurrence, follow-up imaging showed no evidence of disease, with the patient continuing immunotherapy treatment.

## Discussion

Cervical cancer is the second most common malignancy in women worldwide, with 20% of cases classified as adenocarcinoma [11]. GCA is a distinct, HPV-independent subtype of cervical adenocarcinoma [3]. In two recent revisions of 38 and 40 cases, the median age was 46-49 years [3,12]. In our case, the patient was 58 years old.

GAS has been associated with specific genetic alterations, including TP53 and STK11 mutations [10]. Lobular endocervical glandular hyperplasia has been described as a possible precursor lesion, particularly in patients with Peutz-Jeghers syndrome [13]; another genetic alteration associated with GCA is Li-Fraumeni syndrome [3].

The most common presenting symptoms of GCA include abdominal pain and abnormal vaginal bleeding or discharge [10,12]. In the present case, the patient was asymptomatic, with normal gynecological examination, negative HPV testing, and normal cervical cytology performed within the year prior to the diagnosis.

Unlike HPV-associated tumors, GCA is frequently missed by conventional screening methods, leading to delayed diagnosis and advanced-stage presentation [14]. Other studies observed that 50% of patients had normal cytology, 33% showed atypical glandular cells, and 17% had atypical cells not otherwise specified [13].

Serum CA 19-9 elevation has been reported in GCA and other mucinous malignancies and may serve as a useful adjunctive marker. In our patient, CA 19-9 was markedly elevated, supporting the suspicion of a mucinous neoplasm.

Imaging studies often reveal abnormal cervical findings, and in some cases, GAS may mimic ovarian or other gynecologic malignancies [13,15].

Histologically, GCA is characterized by mucinous glands with gastric (pyloric) differentiation. Immunohistochemical markers, such as MUC6, HIK1083, CK7, CAIX, PAX8, and CEA, are commonly expressed, while p16, estrogen receptor, and progesterone receptor expression is typically absent or minimal [16-19]. These features are essential for accurate diagnosis and distinction from other mucinous tumors.

Therapeutic strategies do not differ substantially from those used for other types of cervical cancers; however, patients often require multimodal treatment with adjuvant therapy or initial chemoradiotherapy (CCRT). In a recent review by Ehmann S et al., out of 70 patients with GCA, 9% underwent surgery alone, 35% had surgery with adjuvant therapy, 23% had CCRT, 16% chemotherapy alone, and 7% had neoadjuvant CCRT and a hysterectomy [1].

GAS is associated with an unfavorable prognosis, increased recurrence rate, and poor survival status [8,19]. In this case, recurrence occurred one year and three months after the initial treatment. Recent studies have related GCA with poor response to radiotherapy, with a low three-year PFS (progression-free survival) than other histologic types of cervical adenocarcinoma (44.4% vs 66.7%, p <0.05) [20].

In a retrospective study of patients diagnosed with GCA, over 50% of these patients presented with an advanced stage [4], as is the case with our patient.

## Conclusions

Gastric-type endocervical adenocarcinoma is a rare type of cervical cancer that is not associated with human papillomavirus infection. GCA has a different clinical presentation, and the diagnosis of patients affected by GCA is difficult because HPV is negative, and vaginal cytology is not sensitive. The CA 19.9 tumor marker and CT scan may be useful in cases of suspected GCA. Histological diagnosis should be performed by an expert pathologist. Mucinous tumors, such as GCA, have positive immunohistochemical staining for CK7 and CK20 and elevated Ki67. Most patients affected by GCA require adjuvant therapy due to the late diagnosis and the more aggressive behavior of this tumor. GCA is associated with a poor prognosis, higher recurrence rate, and a low survival rate. Multicenter studies are needed to improve the diagnosis and treatment of these rare tumors.
